# Supplementary material for: Broad Spectrum Functional Activity of Structurally Related Monoanionic Au(III) Bis(Dithiolene) Complexes
Source: Int J Mol Sci. 2022 Jun 27;23(13):7146. doi: 10.3390/ijms23137146 (PMC9266914; doi:10.3390/ijms23137146)
Supplement: Supplementary file 1 [file ijms-23-07146-s001.zip › ijms-1761428-supplementary.pdf]

SUPPLEMENTARY INFORMATION

Broad spectrum functional activity of structurally related monoanionic Au(III) bis(dithiolene) complexes

Yann Le Gal[1], Agathe Filatre-Furcate[1], Dominique Lorcy[1]*, Olivier Jeannin[1], Thierry Roisnel[1] , Vincent Dorcet[1], Diana Fontinha[2], Denise Francisco[2], Miguel Prudêncio[2], Marta Martins[2], Catarina Soeiro[3,4], Sílvia A. Sousa[3,4], Jorge H. Leitão[3,4], Tânia S. Morais[5], Inês Bártolo[6], Nuno Taveira[6], Joana F. Guerreiro[7], Fernanda Marques[7]*

[1] Univ Rennes, CNRS, ISCR (Institut des Sciences Chimiques de Rennes) – UMR 6226, F-35000 Rennes, France

[2] Instituto de Medicina Molecular João Lobo Antunes, Faculdade de Medicina, Universidade de Lisboa, Avenida Professor Egas Moniz, 1649-028 Lisboa, Portugal.

[3] iBB-Institute for Bioengineering and Biosciences, Departmento de Bioengenharia, Instituto Superior Técnico, Universidade de Lisboa, Av. Rovisco Pais, 1049-001 Lisboa, Portugal.

[4] Associate Laboratory, i4HB—Institute for Health and Bioeconomy at Instituto Superior Técnico, Universidade de Lisboa, Av. Rovisco Pais, 1049-001 Lisboa, Portugal

[5] Centro de Química Estrutural and Departamento de Química e Bioquímica, Faculdade de Ciências, Universidade de Lisboa, Campo Grande, 1749-016 Lisboa, Portugal.

[6] iMed.ULisboa, Faculdade de Farmácia da Universidade de Lisboa, Avenida das Forças Armadas 1600-083 Lisboa, Portugal

[7] Centro de Ciências e Tecnologias Nucleares and Departamento de Ciências e Tecnologias Nucleares, Instituto Superior Técnico, Universidade de Lisboa, Estrada Nacional 10 (km 139,7), 2695-066 Bobadela LRS, Portugal.

*Corresponding Authors:

Dominique.lorcy@univ-rennes1.fr

Fmarujo@ctn.tecnico.ulisboa.pt

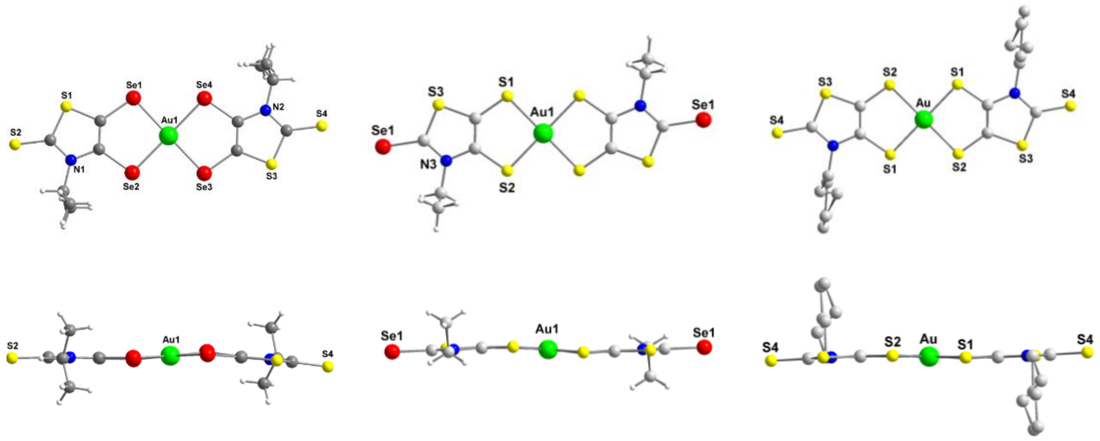

Figure S1. Molecular structures view (top) and side view (bottom) of the monoanionic gold complexes $[AuSeiPr(C{=}S)]^{-1}$ (left), $[AuSEt(C{=}Se)]^{-1}$(middle) and $[AuSBu(C{=}S)]^{-1}$(right).

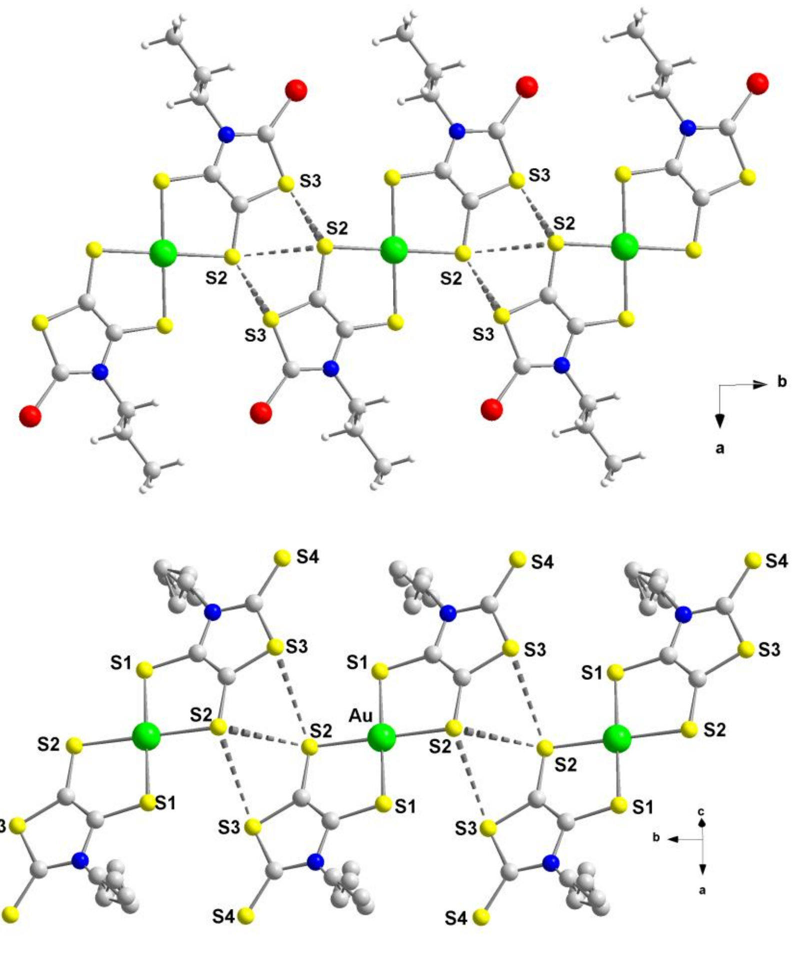

Figure S2 View of the S•••S short contacts between neighbouring monoanionic gold complexes in [**P**][AuSPr(=Se)] (top) and [**P**][AuSBu(=S)] (bottom).

As observed for the other monoanionic gold complexes, short lateral chalcogen•••chalcogen contacts are observed for [**P**][AuSPr(=Se)] and [**P**][AuSBu(=S)]. For [**P**][AuSPr(=Se)], the monoanions form layers along the b axis with several short S••• S interactions shorter than twice the van der walls radii of the sulfur atoms, S2•••S2 3.27 Å, S2•••S3 3.37 Å (vdW S•••S 3.60). Similarly, the monoanions in [**P**][AuSBu(=S)] form layers along the b axis with two short S••• S contacts between neighbouring complexes, shorter than twice the van der walls radii of the sulfur atoms, S2•••S2 3.30 Å, S2•••S3 3.44 Å (vdW S•••S 3.60).

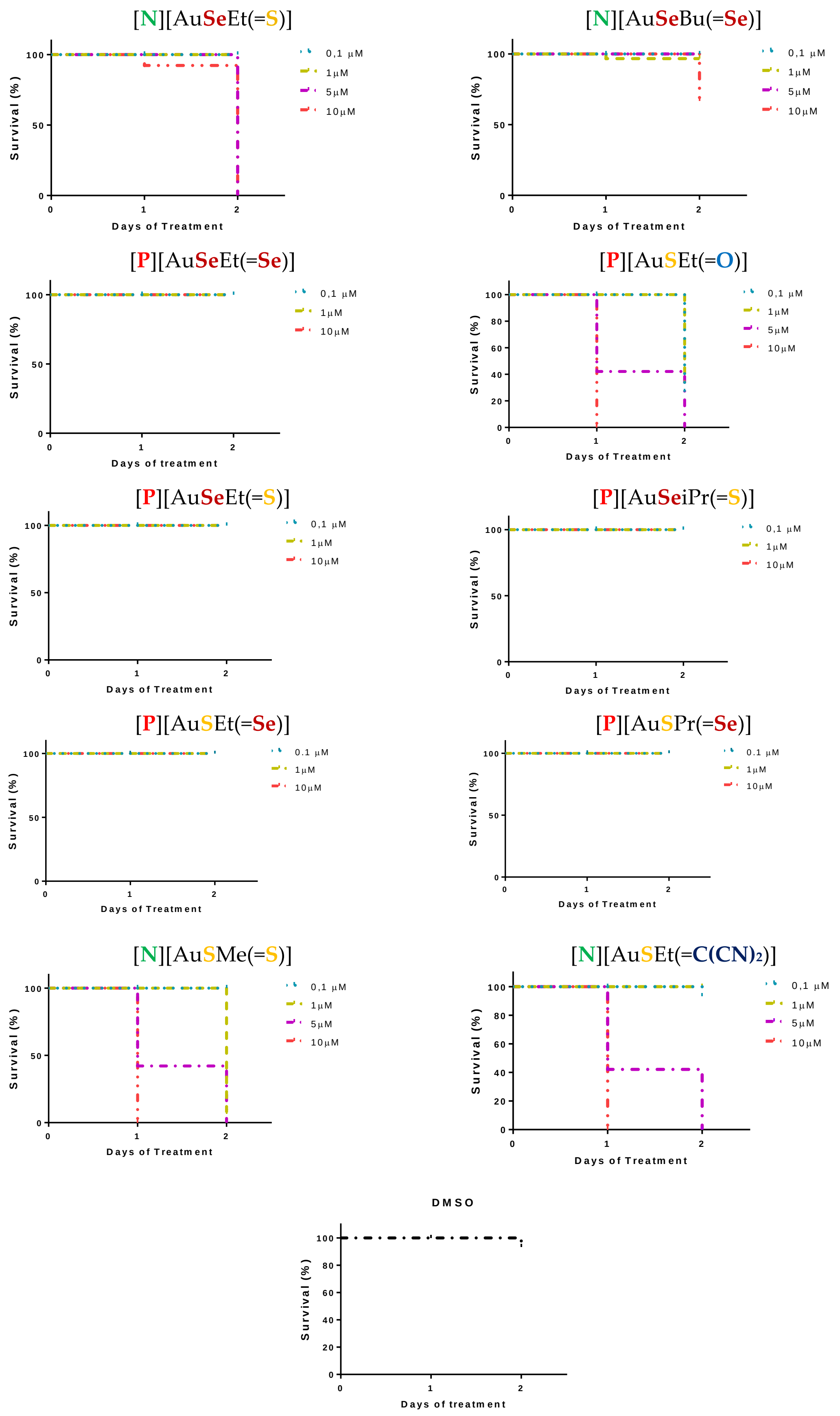

Figure S3. Zebrafish lethality curves of the acute toxicity assay at 48 h for selected Au(III) bis(dithiolene/diselenolene) complexes and the negative control DMSO.

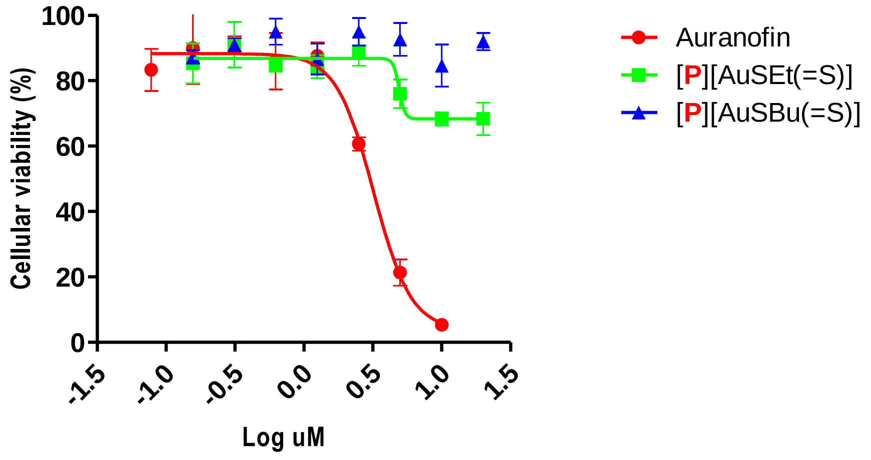

Figure S4. Cytotoxicity of Auranofin, [P][AuSEt(=S)] and [P][AuSBu(=S)] in TZM-bl cells.

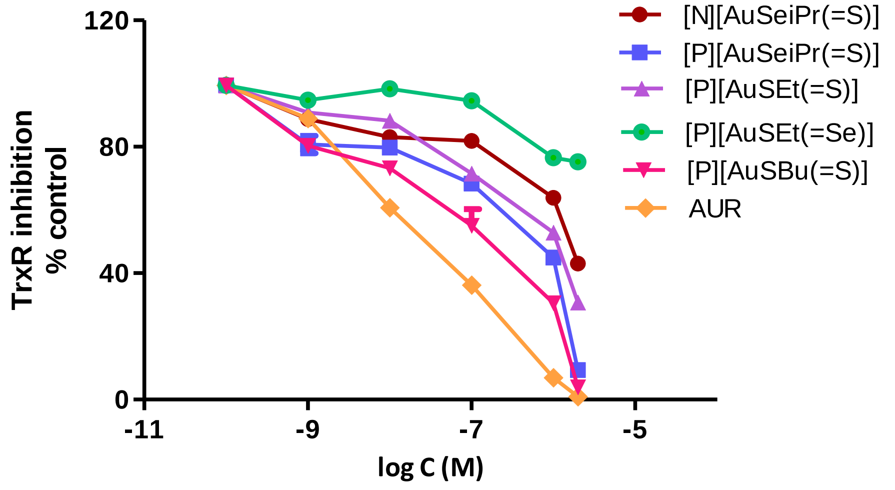

Figure S5. Inhibition of TrxR by the selected gold complexes and Auranofin (AUR).

Table S1. Inhibition of TrxR. IC_{50} values calculated for the selected gold complexes

Complexes	IC_{50} (µM)
[**N**][AuSe*i*Pr(=S)]	1.50 ± 0.50
[**P**][AuSe*i*Pr(=S)]	0.40 ± 0.25
[**P**][AuSEt(=S)]	0.86 ± 0.32

[P][AuSEt(=Se)]	4.81 ± 1.02
[P][AuSBu(=S)]	0.13 ± 0.08
AUR	0.026 ± 0.01

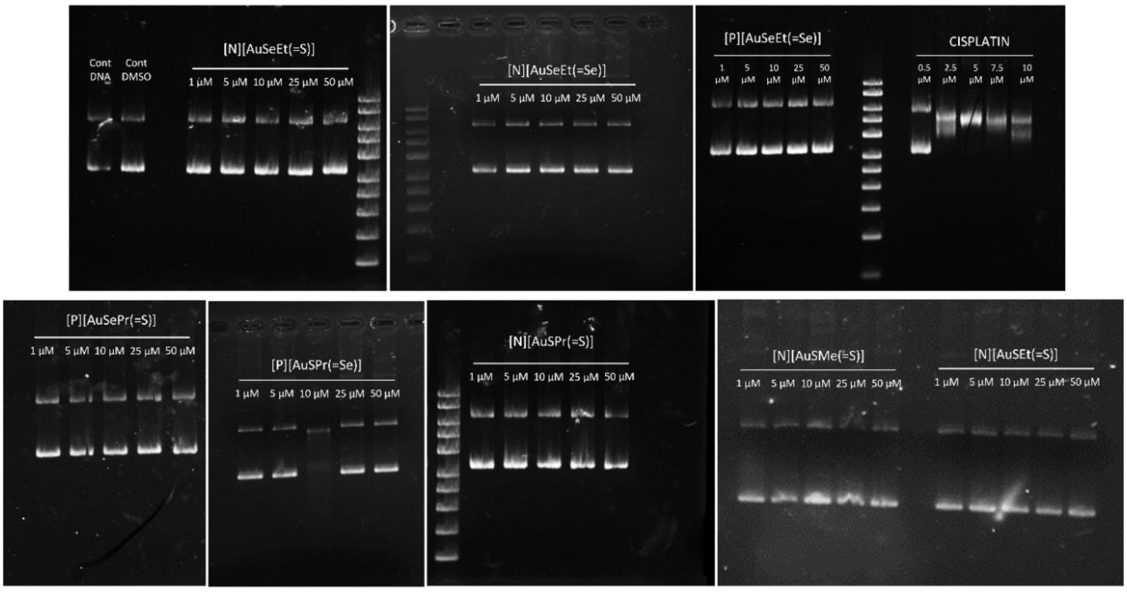

Figure S6. Effect of the selective complexes and cisplatin on the electrophoretic mobility of ϕX174 DNA after 24 h of incubation at 37 °C. Cisplatin was able to alter the electrophoretic mobilities of the nicked and supercoiled forms of DNA in a dose-dependent way but not the gold complexes under study.

Gold complexes-HSA Interaction studies

The mechanisms of fluorescence quenching are typically classified as static or dynamic. Both require molecular contact among the quencher and the fluorophore. Both processes can be described by Stern-Volmer equations. In many cases, the fluorophore is quenched by a combined dynamic and static mechanism, the Stern-Volmer equation [J. R. Lakowicz, Principles of fluorescence spectroscopy, Springer, 2006] for a combined mechanism is:

$$\frac{F_0}{F} = 1 + (K_D + K_S)[Q] + K_D K_S [Q]^2 \qquad \text{(S1)}$$

where F_0 and F are the fluorescence intensities of HSA in the absence and presence of a quencher (Au complex), respectively; K_D and K_S represents the dynamic and static constants, respectively, and [Q] is the quencher concentration. When the fluorescence intensity quenching occurs by a purely static or dynamic mechanism, the following Stern-Volmer equation can be used:

$$\frac{F_0}{F} = 1 + K_q \tau_0 [Q] = 1 + K_{SV}[Q] \qquad \text{(S2)}$$

where K_q, τ_0 and K_{SV} represents the bimolecular quenching constant, the fluorophore lifetime in the absence of the quencher and the Stern-Volmer quenching constant, respectively.

The relationship between the fluorescence intensity of the protein and the complex concentration is given by the equation:

$$log\left(\frac{F_0 - F}{F}\right) = \log K_a + n \log[Q] \qquad \text{(S3)}$$

where K_a is the association constant for a site and n the number of binding sites [Structural analysis and binding domain of albumin complexes with natural dietary supplement humic acid. J. Lumin. 131:2244–2251. doi:10.1016/j.jlumin.2011.06.005].

Table S1. Values of Stern Volmer quenching constant (K_{sv}), association constant (K_a) and number of binding sites (n) for the interaction for HSA with gold compounds.

Compound	K_{SV} (x 10^5 L mol^{-1})	Kq (x 10^{13} L mol^{-1} s^{-1})	R^2	Ka (x 10^4 L mol^{-1})	n	R^2
[P][AuSEt(=S)]	6.48 ± 0.06	6.48 ± 0.06	0.922	1.12 ± 0.02	1.179	0.9946

[P][AuSEt(=Se)]	5.00 ± 0.17	5.00 ± 0.17	0.9931	0.52 ± 0.03	2.135	0.9925
[P][AuSeiPr(=S)]	3.63 ± 0.04	3.63 ± 0.01	0.9906	1.52 ± 0.01	0.957	0.9949
[P][AuSBu(=S)]	14.9 ± 0.13	14.9 ± 0.05	0.9930	9.34 ± 0.01	0.738	0.9906

Table S2. Crystallographic data for monoanionic gold complexes

	[P][AuSePr(=S)]. 2CH3CN	[P][AuSPr(=Se)]. 2CH3CN	[P][AuSBu(=S)]
Formulae	$C_{40}H_{40}AuN_4PS_4Se_4$	$C_{40}H_{40}AuN_4PS_6Se_2$	$C_{38}H_{38}AuN_2PS_8$
FW (g.mol^{-1})	1248.78	1154.98	1007.12
System	monoclinic	monoclinic	monoclinic
Space group	C2/c	C2/c	C2/c
a (Å)	26.7270(7)	27.092(2)	27.9213(12)
b (Å)	7.5774(2)	7.3759(6)	7.2795(3)
c (Å)	24.7399(6)	24.534(2)	23.5768(11)
α (deg)	90	90	90
β (deg)	114.587(1)	114.730(3)	123.2700(10)
γ (deg)	90	90	90
V (Å^3)	4556.1(2)	4453.0(6)	4006.6(3)
T (K)	150(2)	150(2)	293(2)
Z	4	4	4
D_{calc} (g.cm^{-1})	1.821	1.723	1.67
μ (mm^{-1})	6.676	5.290	4.160
Total refls	20486	18627	16097
Abs corr	multi-scan	multi-scan	multi-scan
Uniq refls (R_{int})	4601 (0.0336)	5112 (0.0949)	4580 (0.0584)
Uniq refls (I > 2σ(I))	1628	3358	3467
R_1, wR_2	0.0231, 0.0708	0.0604, 0.1366	0.0423, 0.0919
R_1, wR_2 (all data)	0.0319, 0.0911	0.1028, 0.1584	0.064, 0.1025
GOF	1.238	1.001	1.018

	[P][AuSEt(=Se)]. 2CH3CN	[P][AuSeiPr(=S)]	[P][AuSEt(=C(CN)2)]
Formulae	$C_{38}H_{36}AuN_4PS_6Se_2$	$C_{36}H_{34}AuN_2PS_4Se_4$	$C_{40}H_{30}AuN_6PS_6$
FW (g.mol^{-1})	1126.92	1166.67	1014.99
System	monoclinic	monoclinic	triclinic
Space group	C2/c	P2$_1$/n	P-1
a (Å)	27.4946(17)	10.7523(4)	9.4257(4)
b (Å)	7.4358(5)	16.6504(6)	13.9527(6)
c (Å)	24.6489(19)	22.2538(10)	16.1706(6)
α (deg)	90	90	76.297(2)
β (deg)	119.207(3)	99.572(2)	88.975(2)
γ (deg)	90	90	75.146(2)
V (Å^3)	4398.6(5)	3928.6(3)	1995.00(14)
T (K)	150(2)	150(2)	150(2)
Z	4	4	2
D_{calc} (g.cm^{-1})	1.702	1.972	1.690
μ (mm^{-1})	5.353	7.733	4.080

Total refls	18201	28608	37161
Abs corr	multi-scan	multi-scan	multi-scan
Uniq refls (R_{int})	4982 (0.0344)	8797 (0.0331)	9149 (0.0430)
Uniq refls ($I > 2\sigma(I)$)	4245	7095	8115
R_1, wR_2	0.024, 0.0554	0.0393, 0.0905	0.0352, 0.0714
R_1, wR_2 (all data)	0.0315, 0.0583	0.0553, 0.0968	0.0433, 0.0745
GOF	1.039	1.034	1.054

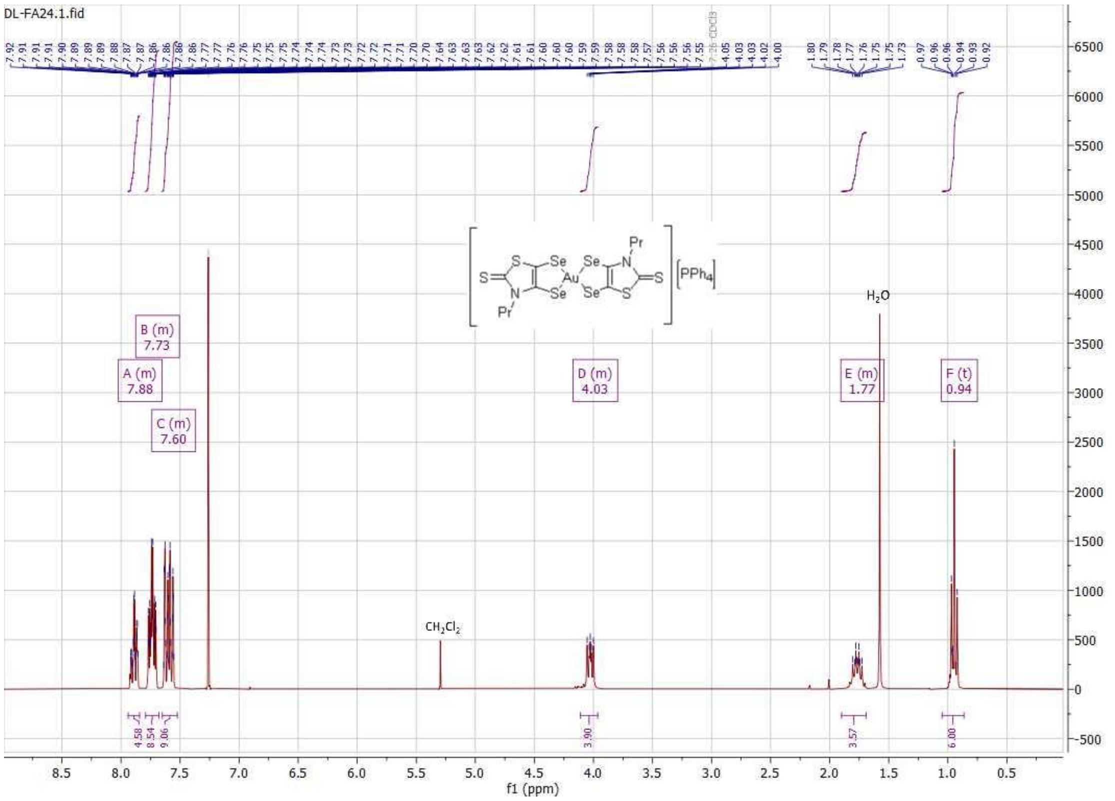

Figure S7. ^{1}H NMR spectrum of **[P][AuSePr(=S)]** in $CDCl_3$

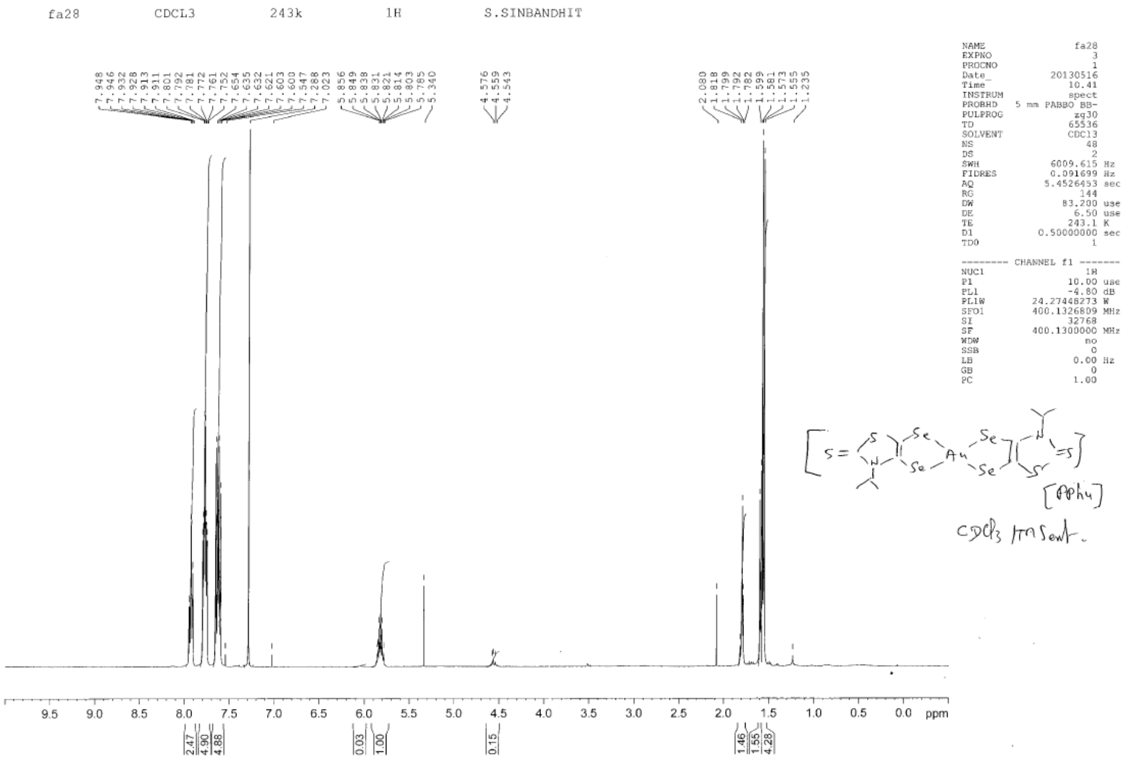

Figure S8. ^{1}H NMR spectrum at 243Kof **[P][AuSe*i*Pr(=S)]** in $CDCl_3$

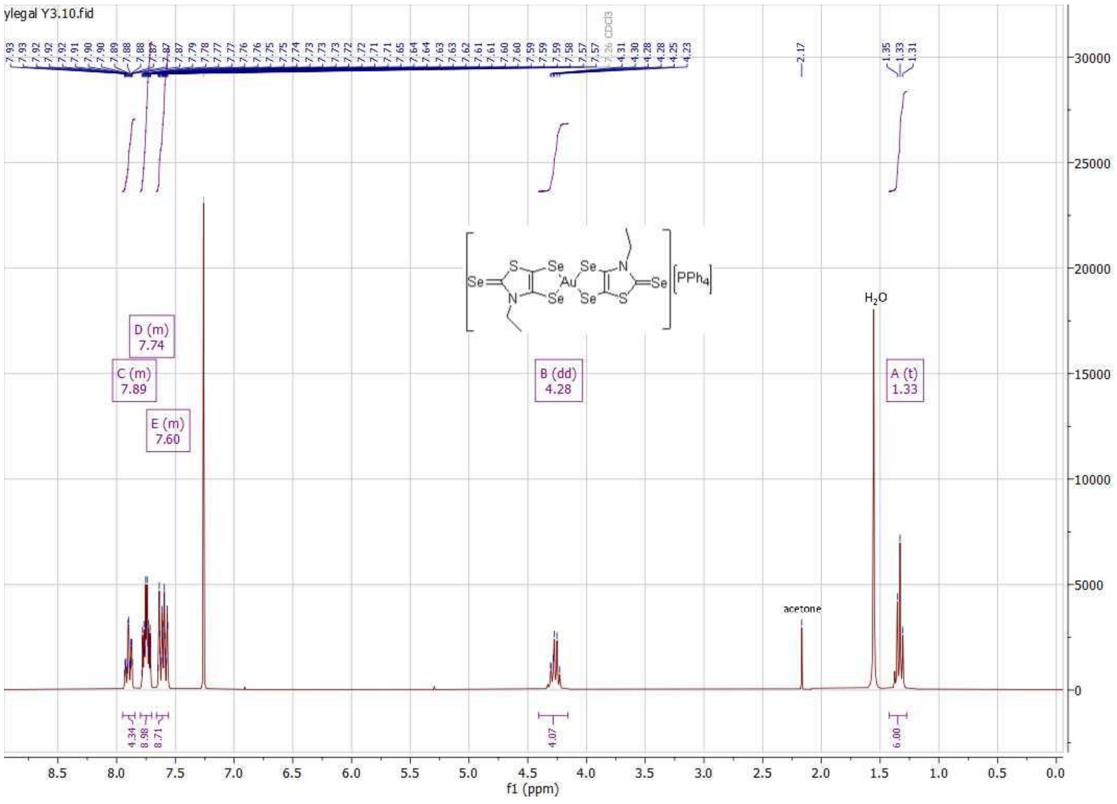

Figure S9. ^{1}H NMR spectrum of **[P][AuSeEt(=Se)]** in $CDCl_3$

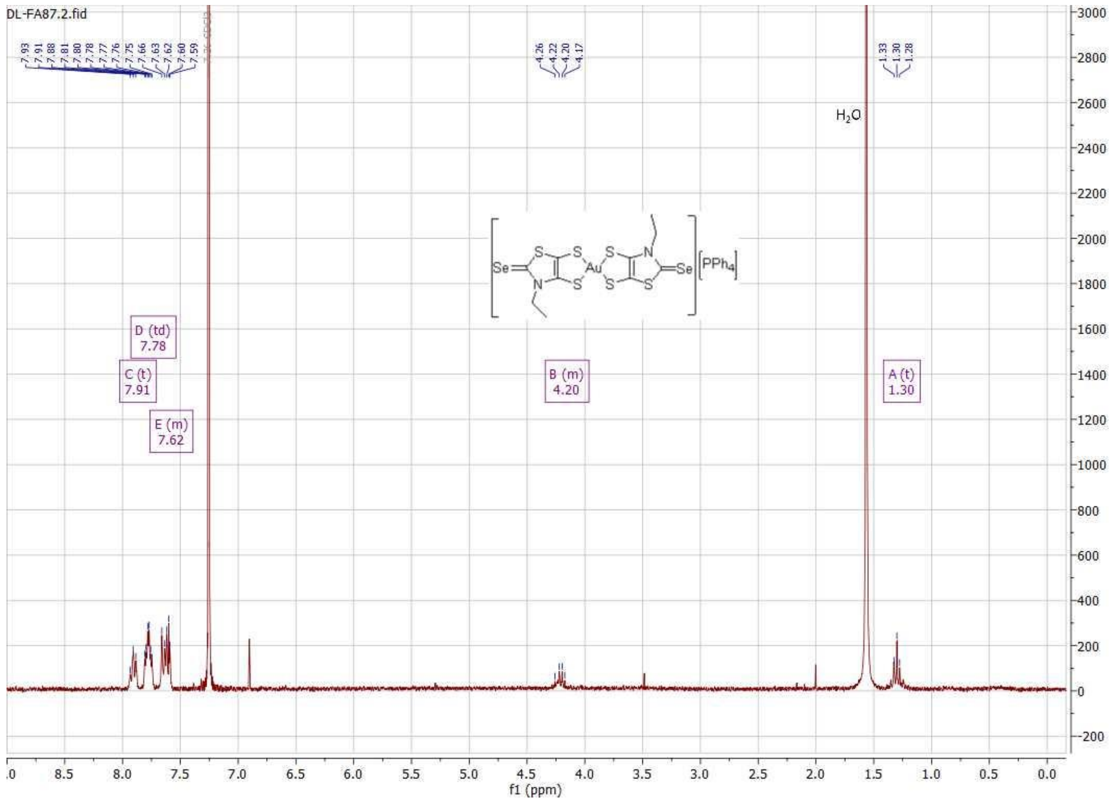

Figure S10. ^{1}H NMR spectrum of **[P][AuSEt(=Se)]** in $CDCl_3$

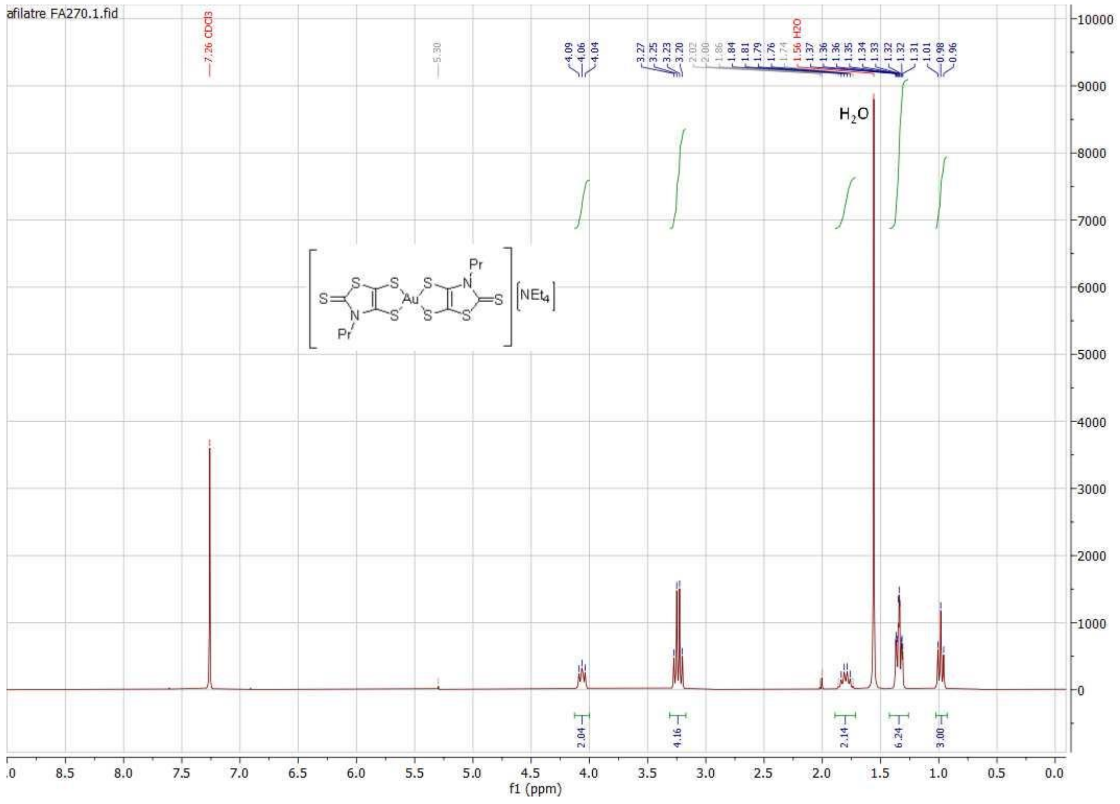

Figure S11. ^{1}H NMR spectrum of **[N][AuSPr(=S)]** in $CDCl_3$

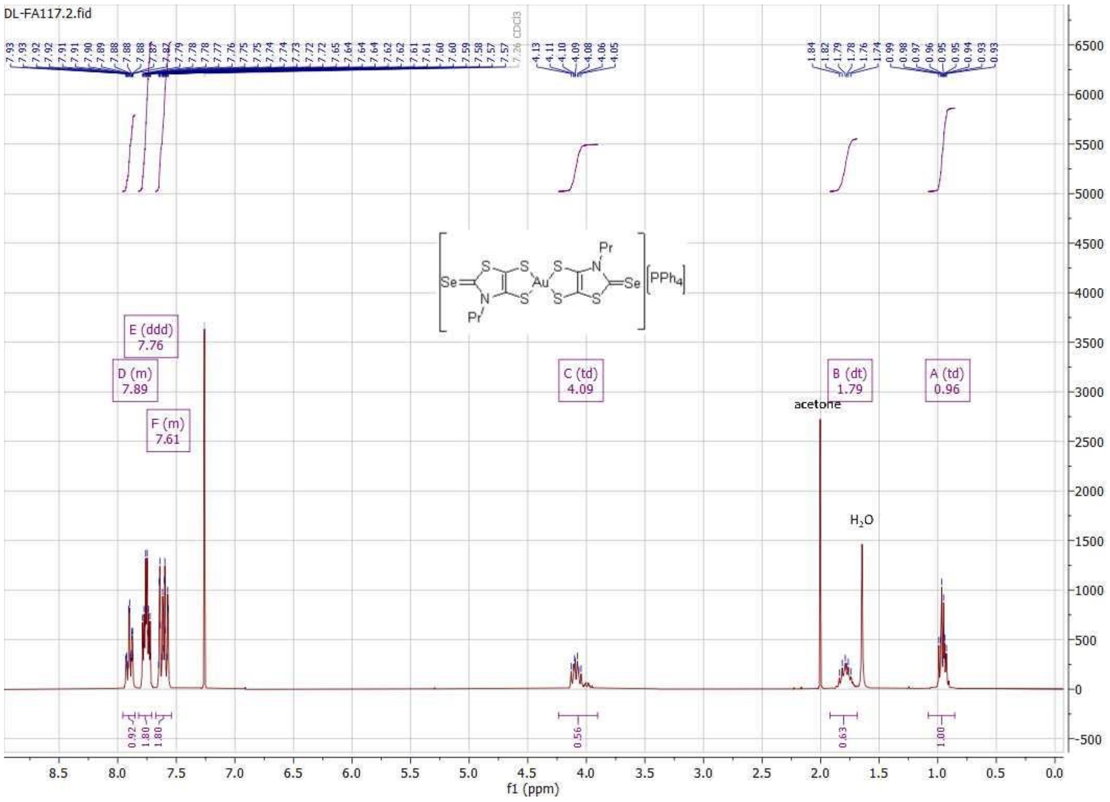

Figure S12. ^{1}H NMR spectrum of **[P][AuSPr(=Se)]** in $CDCl_3$

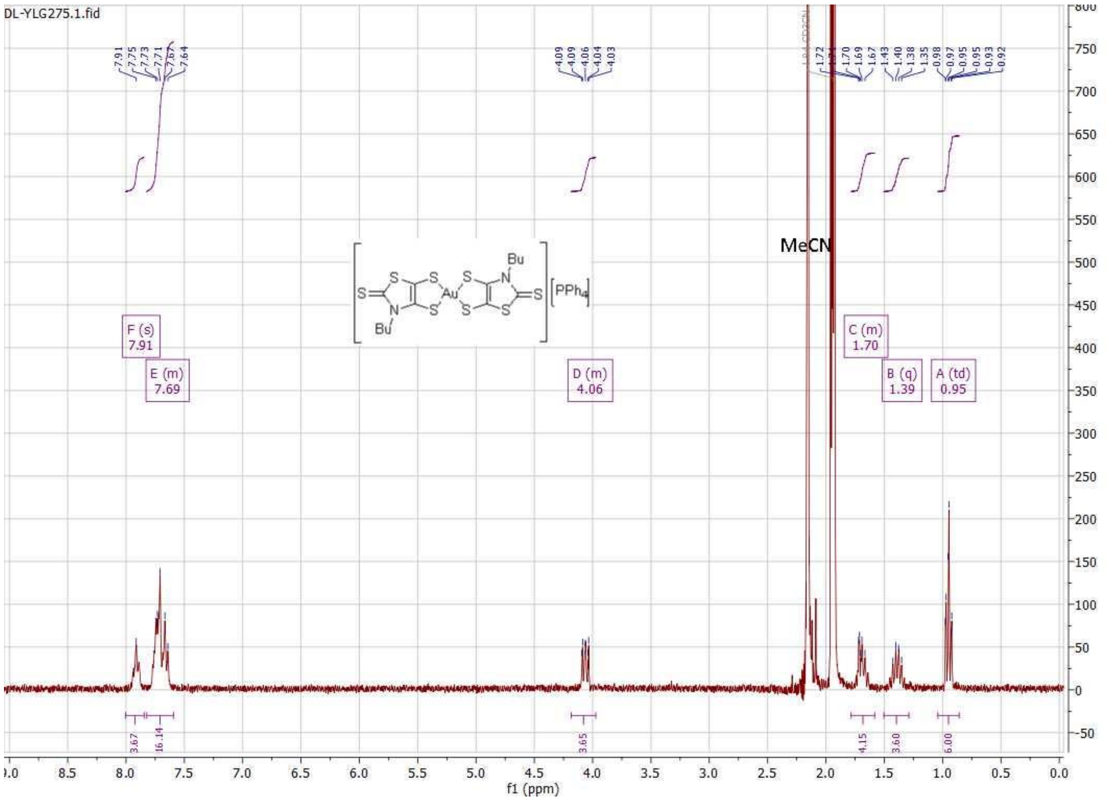

Figure S13. ^{1}H NMR spectrum of **[P][AuSBu(=S)]** in CD_3CN

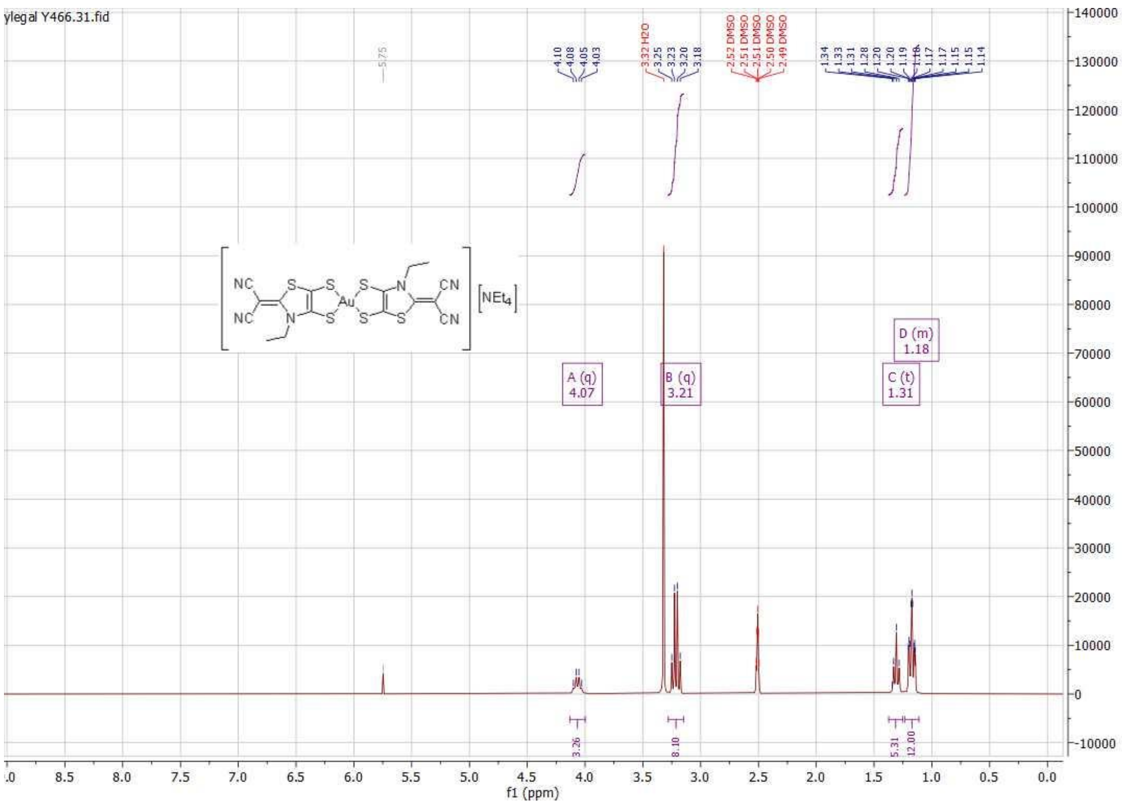

Figure S14. ^{1}H NMR spectrum of **[N][AuSEt(=C(CN)$_2$)]** in $(CD_3)_2SO$

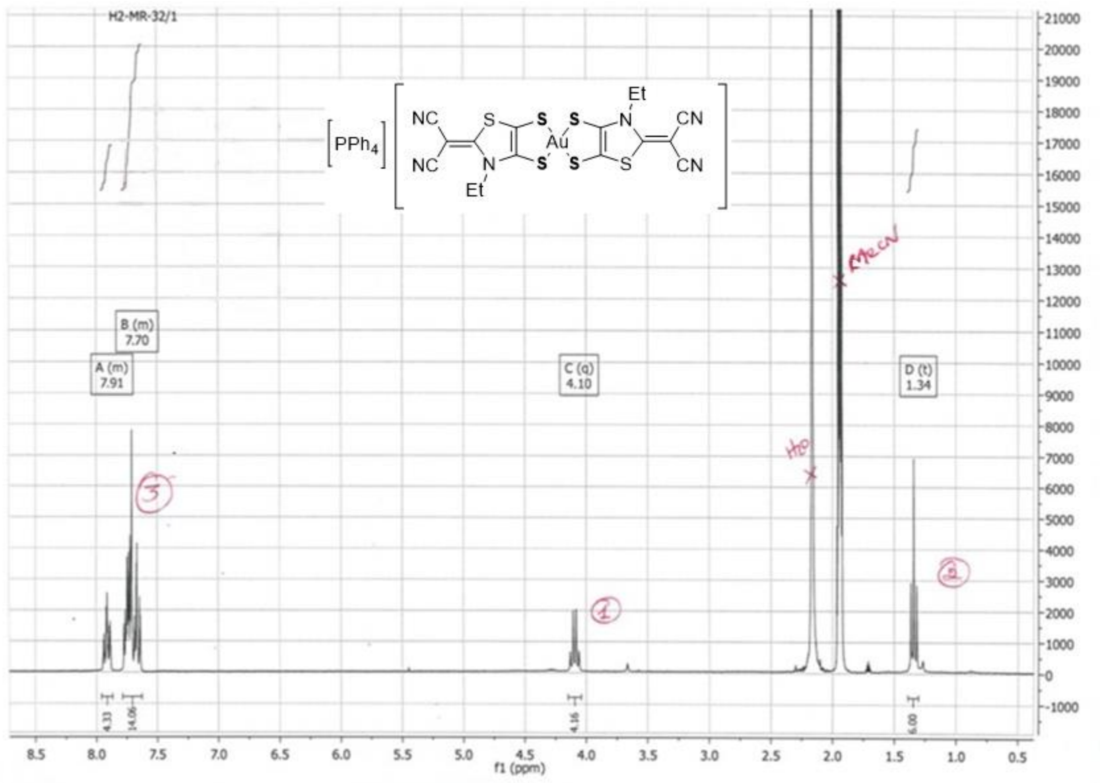

Figure S15. ^{1}H NMR spectrum of **[P][AuSEt(=C(CN)$_2$)]** in CD_3CN
